# Case Report: Unexpected Remission From Extreme and Enduring Bulimia Nervosa With Repeated Ketamine Assisted Psychotherapy

**DOI:** 10.3389/fpsyt.2021.764112

**Published:** 2021-11-17

**Authors:** Anya Ragnhildstveit, Laura Kate Jackson, Sarah Cunningham, Linda Good, Quinn Tanner, Matthew Roughan, Patricia Henrie-Barrus

**Affiliations:** ^1^Department of Psychology, University of Utah, Salt Lake City, UT, United States; ^2^Behavioral Science Department, Utah Valley University, Orem, UT, United States; ^3^Division of Public Health, University of Utah School of Medicine, Salt Lake City, UT, United States; ^4^Marriage and Family Therapy Program, Capella University, Minneapolis, MN, United States; ^5^Riverwoods Behavioral Health, Provo, UT, United States; ^6^Department of Educational Psychology, University of Utah, Salt Lake City, UT, United States

**Keywords:** bulimia nervosa, eating disorder, binge-eating, purging, ketamine, ketamine assisted psychotherapy, psychopharmacology, case report

## Abstract

**Background:** Bulimia nervosa is a disabling psychiatric disorder that considerably impairs physical health, disrupts psychosocial functioning, and reduces overall quality of life. Despite available treatment, less than half of sufferers achieve recovery and approximately a third become chronically ill. Extreme and enduring cases are particularly resistant to first-line treatment, namely antidepressants and cognitive behavioral therapy, and have the highest rate of premature mortality. Here, we demonstrate that in such cases, repeated sessions of ketamine assisted psychotherapy (KAP) is an effective treatment alternative for improving symptoms.

**Case Presentation:** A 21-year-old woman presented with extreme and enduring bulimia nervosa. She reported recurrent binge-eating and purging by self-induced vomiting 40 episodes per day, which proved refractory to both pharmacological and behavioral treatment at the outpatient, residential, and inpatient level. Provided this, her physician recommended repeated KAP as an exploratory and off-label intervention for her eating disorder. The patient underwent three courses of KAP over 3 months, with each course consisting of six sessions scheduled twice weekly. She showed dramatic reductions in binge-eating and purging following the first course of treatment that continued with the second and third. Complete cessation of behavioral symptoms was achieved 3 months post-treatment. Her remission has sustained for over 1 year to date.

**Conclusions:** To our knowledge, this is the first report of repeated KAP used to treat bulimia nervosa that led to complete and sustained remission, a rare outcome for severe and enduring cases, let alone extreme ones. Additionally, it highlights the degree to which KAP can be tailored at the individual level based on symptom severity and treatment response. While its mechanism of action is unclear, repeated KAP is a promising intervention for bulimia nervosa that warrants future research and clinical practice consideration.

## Introduction

Bulimia nervosa (BN) is a disabling psychiatric disorder characterized by recurrent binge-eating (consuming objectively large amounts of food with a sense of lost control) and inappropriate compensatory behaviors (self-induced vomiting; laxative, diuretic, or medication misuse; and fasting or excessive exercise) aimed at preventing weight gain (1, 2). Overtime, the severity of these patterns significantly disrupts physical health and psychosocial functioning, as well as impacts families and communities at large (3). Approximately 50 million people worldwide will develop BN at some point in their life (4). Moreover, studies have found BN to be associated with concomitant psychiatric comorbidity [e.g., mood disorders and substance abuse; (5, 6)] in addition to premature mortality due to medical complications (7–9). Death by suicide is also eight times more likely to occur among individuals with BN compared to the general population, with more than a third experiencing lifetime rates of non-suicidal self-injury (10, 11).

While pharmacological (e.g., selective serotonin reuptake inhibitors) and behavioral (e.g., cognitive behavioral therapy) interventions are effective in managing BN (12, 13), many individuals do not respond to first-line treatment, are unsuccessful in later attempts, and fail to change over protracted periods (14, 15). Nearly 30% of sufferers become chronically ill as a result (16). For such chronic refractory cases, the paucity of evidence-based treatments has prompted paradigm shifts toward harm reduction and palliative care *over* recovery (17, 18).

Ketamine, a non-competitive N-methyl-D-aspartate receptor (NMDAr) antagonist, is an emerging therapy for treatment-resistant mood disorders (19, 20). Single-dose studies have consistently shown rapid antidepressant and anti-suicidal effects following ketamine treatment, though are relatively short-lived (1–4 weeks) (21–28). Ketamine assisted psychotherapy (KAP) has therefore been utilized to prolong ketamine's efficacy and maximize therapeutic outcomes (29–34). To date, few studies have used ketamine for the treatment of eating disorders, including one open-label study (35), two case reports (36, 37), and one longitudinal case series (38), all of which administered ketamine without a psychotherapeutic component. Nonetheless, the results are encouraging. Here, we report the case of a young woman suffering from extreme and enduring BN, according to CARE (CAse REport) guidelines (39), who demonstrated remarkable symptom improvement following repeated sessions of KAP.

## Case Presentation

A 21-year-old woman with BN of 9 years presented to the outpatient clinic, Forum Health. She was first diagnosed with BN, binge-eating/purging type, at 12.5 years of age to which the severity of her symptoms steadily increased overtime. On presentation, she reported alarming rates of binge-eating and purging by self-induced vomiting, averaging ~40 episodes per day for the last 12 months. Based on this frequency, her BN was categorized as “extreme” according to *Diagnostic and Statistical Manual of Mental Disorders, Fifth Edition* (DSM-5) criterion (14 or more episodes per week). Clinical assessment and scoring on the Eating Disorder Examination Questionnaire [EDE-Q; (40, 41)] additionally confirmed the severity of her illness. No laxative or diuretic abuse was reported. While not active in psychiatric treatment, the patient was taking potassium chloride 20 mEq extended-release twice daily for hypokalemia as well as trazodone 100 mg once daily in the evenings for sleep. At 161.92 cm tall and 46.26 kg in weight [body mass index (BMI) = 17.6 kg per m^2^], the patient was amenorrheic and described body image disturbances, intense fear of gaining weight, and obsessive-compulsive tendencies around food (counting calories, binging by order of food group, and inability to discard uneaten items). She further displayed pronounced bilateral parotid sialadenosis (enlargement of the salivary glands) and pseudo-idiopathic edema, otherwise known as pseudo-bartter's syndrome (PBS): a rare and painful complication of BN characterized by hyperaldosteronism, metabolic alkalosis, and hypokalemia (42). As a University student studying cognitive neuroscience, the patient was obliged to take a medical leave due functional decline. “I lost all ability to take care of myself. I could not think clearly or show up for classes. I stopped socializing and running errands. I could hardly maintain basic hygiene.”

Her psychiatric history included an adolescent sexual assault by a treating physician (13 years of age [2011]); a suicide attempt by cut throat injury at the level of the hyoid bone, which required emergency transportation and thyroid cartilage repair as well as inpatient hospitalization (13 years of age [2011]); a second suicide attempt by drug overdose involving mixed opioids, barbiturates, and antidepressants that resulted in emergency room hospitalization (15 years of age [2013]); and a blitz rape (surprise attack) by an unknown assailant (19 years of age [2017]). The patient's history also contained reports of major depression, general anxiety, and obsessive-compulsive disorder. There was no family history of eating disorders, including BN.

As an outpatient, she was treated with various pharmacotherapies (fluoxetine 40 mg once daily, citalopram 20 mg once daily, and naltrexone 50 mg twice daily), behavioral interventions (cognitive behavioral therapy, mindfulness-based stress reduction, and eye movement desensitization and reprocessing), and nutritional counseling (dietary modification and time-based feeding). She additionally was prescribed spironolactone 25 mg twice daily, a potassium-sparing diuretic, on multiple occasions to treat PBS following attempts at purging cessation. However, the patient's binge-purge patterns continued. Finally, she received inpatient, residential, and intensive-outpatient eating disorder care (15–16 years of age [2013–2014]), which the patient described as a “traumatic experience” that resulted in immediate relapse upon discharge.

“My parents pulled me out of class and dropped me off at a center, leaving me there for almost 10 months. It was like being in prison. I was completely cut off from my friends and family. I was forced to eat unreasonable amounts of food at each meal. And I learned new [eating disorder] tricks from other patients that I tried later on. It was not a place conducive to recovery, at least for me. It just made my condition worse.”

Her medical history detailed emergency room hospitalizations for hypokalemia (16, 19, and 20 years of age [2014, 2017, 2018]), gastroesophageal reflux disorder (17–21 years of age [2015–2019]), gastric and duodenal ulcers (19 and 21 years of age [2017, 2019]), hypothyroidism (20–21 years of age [2018–2019]), and adrenocortical insufficiency (20–21 years of age [2018–2019]). Porcelain-laminate veneers were also placed on 10 of her teeth due to dental caries and enamel erosion from chronic purging (21 years of age [2019]).

**Figure 1:**
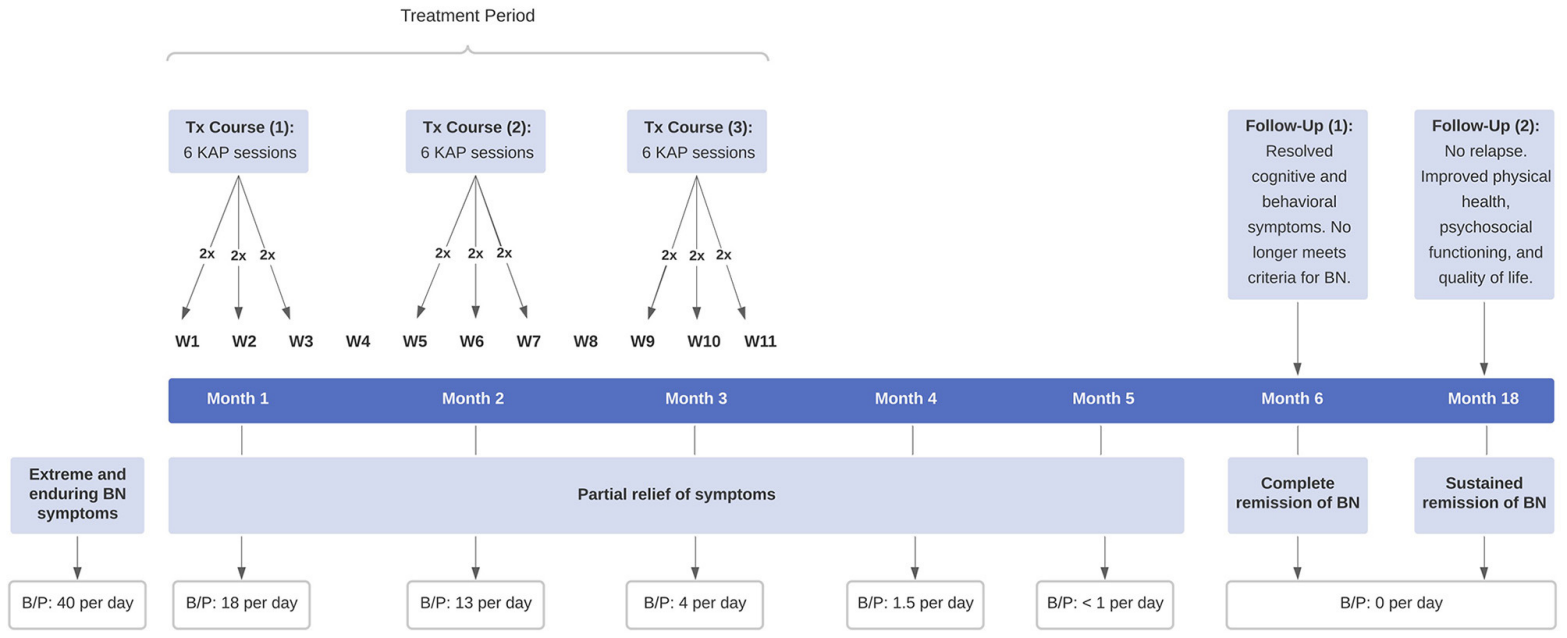
Timeline of clinical events. The patient received three courses of repeated KAP for extreme and enduring BN, consisting of six sessions per course scheduled twice weekly for 3 weeks. KAP, ketamine assisted psychotherapy; BN, bulimia nervosa; B/P, binge-eating and purging.

Given the patient's extreme and chronic refractory state, her physician recommended repeated KAP, with the understanding it constituted an exploratory and off-label intervention for her eating disorder. She consented to treatment following a comprehensive medical evaluation and in-depth review of the procedures, risks, and possible side effects. A signed consent form was obtained. Prior to treatment, she met with a clinical psychologist to establish rapport and therapeutic alliance. The patient then underwent one course of repeated KAP, consisting of six sessions scheduled twice weekly for 3 weeks, with a minimum interval between sessions of 48 h (Figure 1). Each KAP session involved guided psychotherapy combined with racemic ketamine hydrochloride (0.5 mg per kg bodyweight suspended in 0.9% normal saline) administered intravenously over 40 min. The drug regimen was standard practice in the clinic for sub-anesthetic ketamine infusions, which is most commonly used for treating psychiatric disorders and is supported by a substantial body of literature (43, 44). A person-centered, humanistic approach to psychotherapy was employed to facilitate the process of self-actualization and therapeutic change. KAP sessions were preceded by 30 min of preparatory psychotherapy and delivered in a private room with dimmed lights, ambient music, and textile art on the ceiling. The intervention components and ketamine regimen remained the same for all five consecutive sessions; and blood pressure, heart rate, and oxygen saturation were continuously monitored. Due to the severity of her eating disorder, however, the patient returned to the clinic 1 month later for a second course of repeated KAP and then again 1 month later for a third.

**Figure 2:**
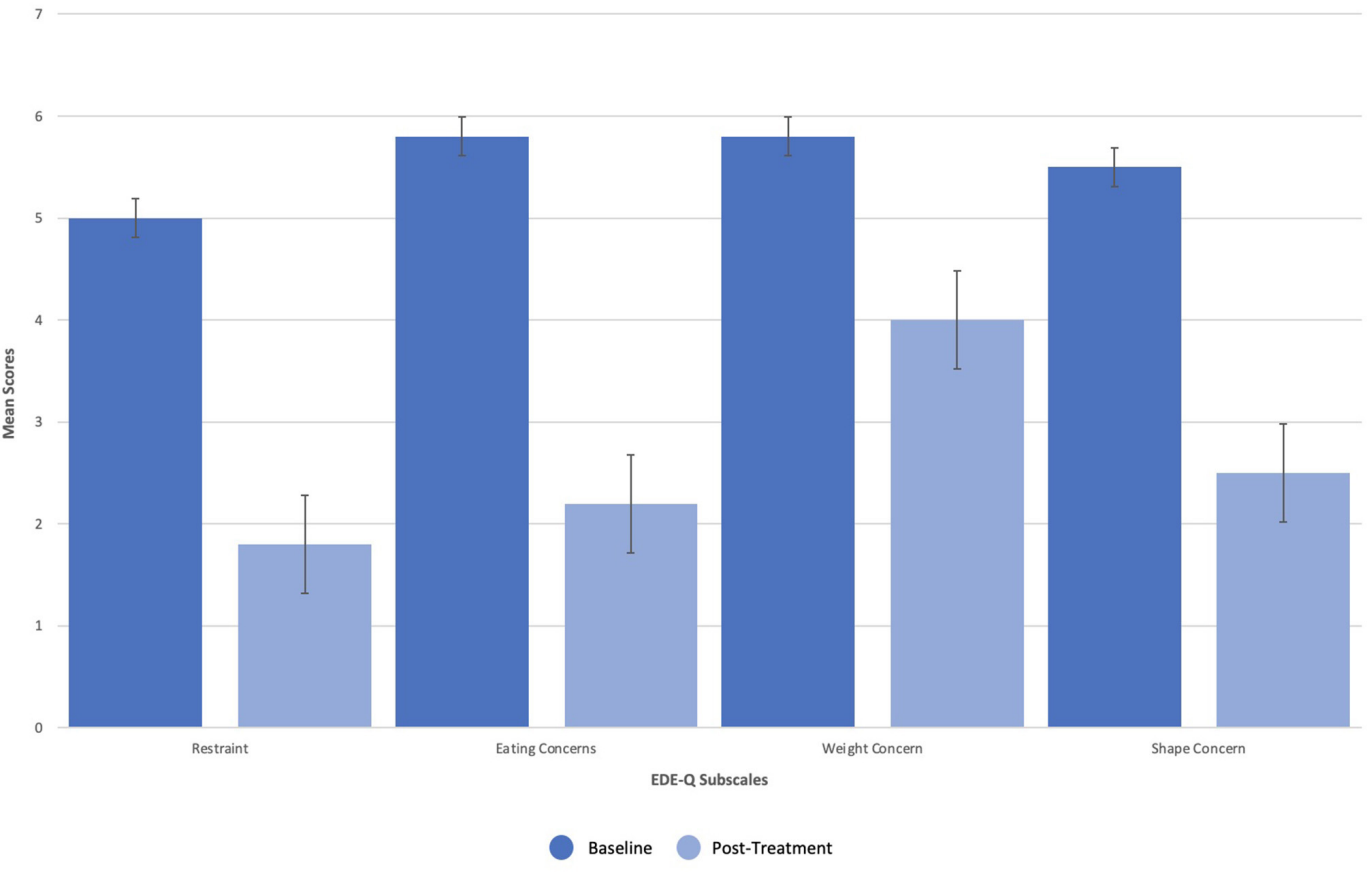
Change in scoring on the Eating Disorder Examination Questionnaire (EDE-Q).

**Figure 3:**
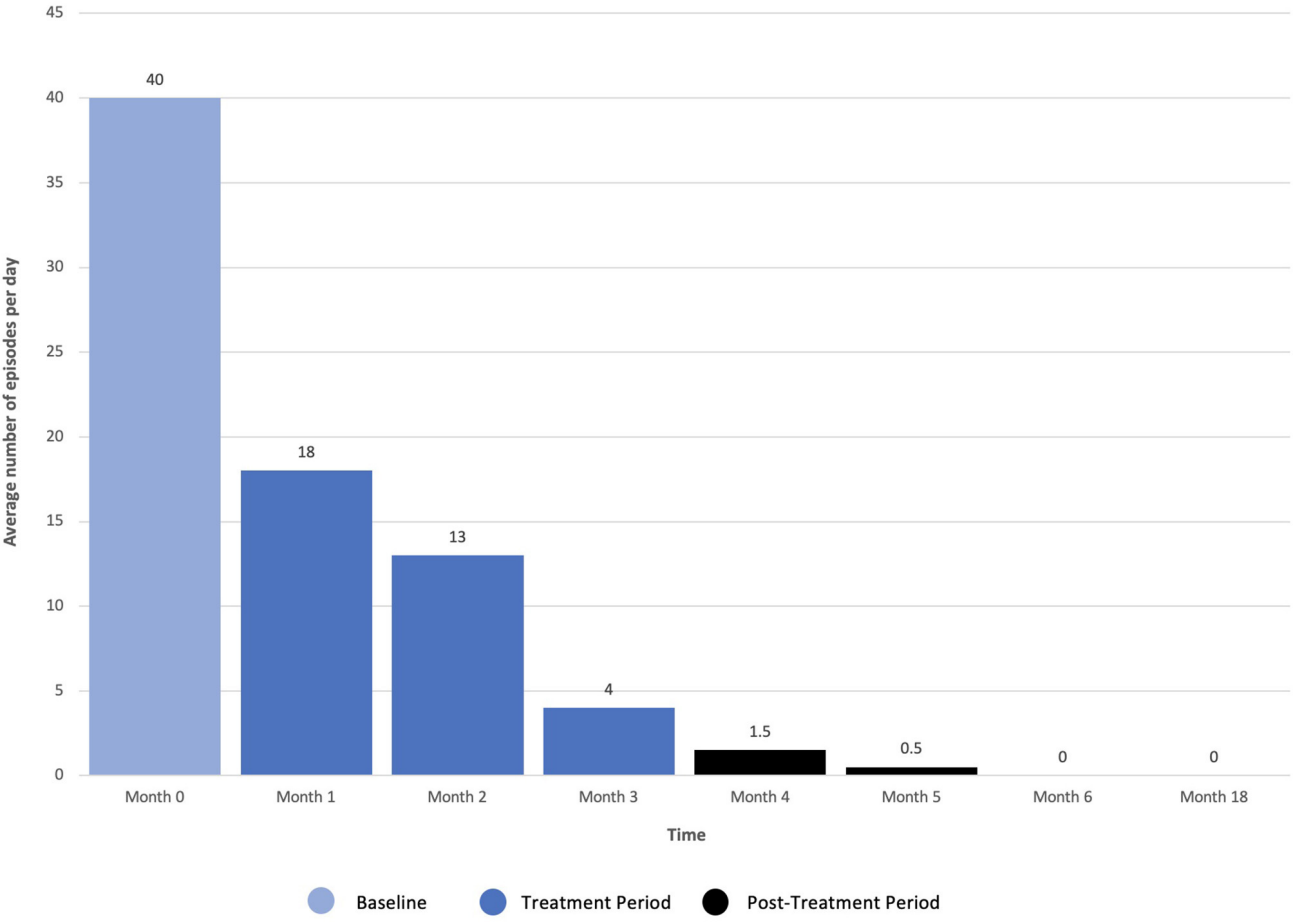
Frequency of daily binge-eating and purging following repeated ketamine assisted psychotherapy.

Dissociation, ego dissolution, and perceptual distortions were present during all KAP sessions, as evidenced by the patient's description of “being disconnected from reality,” “losing [her] sense of identity and self,” and “seeing abstract geometric patterns.” She further exhibited mild diplopia (double vision), nystagmus (involuntary oscillations of the eyes), and alogia (lack of speech) during treatment that resolved completely. No other side effects or adverse events were reported. The patient's eating disorder symptoms remitted over the course of treatments, as measured by change in scoring on the EDE-Q as well as entries from a daily tracking log that recorded frequency of binge-eating and purging. On the EDE-Q, her global score dropped from 31.8 at baseline to 15.0 by the end of all three courses (18 sessions), with similar patterns recorded across all four subscales: “Restraint” (*M* = 5.0, *SD* = 2.2 to *M* = 1.8, *SD* = 1.3), “Eating Concern” (*M* = 5.8, *SD* = 0.5 to *M* = 2.2, *SD* = 1.5), “Weight Concern” (*M* = 5.8, *SD* = 0.5 to *M* = 4.0, *SD* = 1.9), and “Shape Concern” (*M* = 5.5, *SD* = 0.8 to *M* = 2.5, *SD* = 1.6) (Figure 2). Additionally, the patient's tracking log showed decreases in binge-eating and purging from 40 to 18 episodes per day after the first course of treatment (6 sessions), 18 to 13 episodes per day after the second course of treatment (12 sessions), and 13 to 4 episodes per day after the third course of treatment (18 sessions) (Figure 3).

Most notably, the patient stopped her binge-eating and purging behaviors 3 months post-treatment. Given her initial severity and chronic refractory state, this degree of improvement was striking. The patient's daily tracking log additionally showed no signs of relapse 1 year later, accompanied by marked improvement in psychosocial functioning. Specifically, she reported feeling “free” from intrusive BN thoughts and compulsions, “less impulsive” when faced with the urge to binge and purge, and “more confident” about her body in general. The patient has since resumed her academic studies in preparation for a doctoral program.

## Discussion

Severe, chronic, and refractory eating disorder symptoms are unfortunately common among patients with BN. In this case, we describe a young woman with extreme and enduring BN, who remained unresponsive to first-line treatment for nearly a decade, despite care at the outpatient, residential, and inpatient level. Her eating disorder was extreme, insofar as she engaged in recurrent binge-eating and purging by self-induced vomiting 40 episodes per day, which significantly exceeds DSM-5 criterion (14 or more episodes per week). Given the severity of her illness, complete and sustained remission with three courses of repeated KAP (18 sessions) was both remarkable and unanticipated. These findings are more robust provided the patient was not active in psychiatric treatment for 1 year prior to clinic admission, excluding her long-standing prescription of potassium chloride for hypokalemia and trazodone for sleep. If ketamine and psychotherapy act synergistically, with therapy priming and enhancing the response to treatment, then its combined effect may explain the striking improvements in symptoms. Serial treatments likely account for the durability of response necessary for sustained remission, which is consistent with literature (45–48).

Provided this is the first report of repeated KAP used as an exploratory and off-label intervention for BN, it is important to consider the a-priori context. Clinical recommendation to pursue repeated KAP was prompted by three factors. First, the patient's psychiatric and medical history that detailed unsuccessful treatment attempts, including pharmacotherapies, behavioral treatments, and nutritional counseling—even at higher levels of eating disorder care; and significant trauma to which accumulating evidence has shown ketamine to yield positive effects for (49–51). Second, her severe functional impairment in three major life domains, including academic work, social and family engagement, and personal responsibilities. The patient was binge-eating and purging nearly to the exclusion of all other activities, spending more time “alone in the bathroom than with [her] friends or family.” Finally, an open-label case series on repeated ketamine in severe and enduring anorexia nervosa, showing modest improvements in eating disorder symptoms (38).

The patient's impetus for treatment was largely driven by fear of premature mortality—that if she did not attempt something new, she was going to “eat [herself] to death,” quite literally. Serious degradation in the patient's physical and mental health status were particularly motivating. Apart from transient psychological (dissociation, ego dissolution, and perceptual distortions) and physiological (mild diplopia, nystagmus, and alogia) side effects of ketamine that resolved completely after each session, the treatment was well-tolerated. Following all three courses of treatment, the patient dramatically reduced her binge-eating and purging behaviors by 90% compared to baseline, as measured by the EDE-Q and daily tracking logs. She also demonstrated considerable improvements in disordered eating psychopathology that were captured by the subscales of the EDE-Q, most notably “Restraint” (e.g., dietary rules and avoidance of food) and “Eating Concerns” (e.g., preoccupation with calories and fear of losing control over eating). Moreover, the patient regained control of her impulsive eating as well as resolved her obsessive-compulsive neurosis, which align with previous BN-specific findings from a study on intermittent ketamine infusions in eating disorders (35). At 3 months follow-up, she achieved complete cessation of binge-eating and purging and no longer met diagnostic criteria for BN. The magnitude of response neither diminished over time, with no signs of relapse at 15 months follow-up, contrary to studies showing rapid decline of effects after treatment (28, 52, 53). With sustained remission, the patient has adopted a healthier relationship with food, established psychosocial stability in her life, and resumed her academic studies in preparation for graduate school.

This is a single case report with inherent limitations in generalizing the findings to other patients with BN. The lack of polypharmacy or medication washout is an additional limitation that may have unknowingly mediated improvements. Furthermore, it is unclear as to whether ketamine or psychotherapy produced greater clinical benefit, if both are coadjuvant and necessary, or if the treatment would have been as effective without psychotherapy and/or fewer sessions. Finally, a person-centered, humanistic approach to psychotherapy was employed, differing from more traditional methods, such as cognitive-behavioral, interpersonal, and psychodynamic therapy. Open pilot studies as well as fully-powered randomized controlled trials with longitudinal assessment are thus required to establish whether the outcome of this case can be replicated, to what degree ketamine and psychotherapy contribute to the overall success of the treatment, and the comparative efficacy of different psychotherapeutic interventions. Further research is also warranted to optimize KAP duration and frequency for this patient population.

## Conclusions

This study provides compelling evidence that repeated KAP is an effective treatment for extreme and enduring BN, which is exceedingly resistant to first-line therapies and associated with poor prognosis. It further highlights the utility of combined strategies that may prolong ketamine's efficacy, and subsequently maximize therapeutic outcomes at the individual level.

## Data Availability Statement

The original contributions presented in the study are included in the article/supplementary material, further inquiries can be directed to the corresponding author/s.

## Ethics Statement

Ethical review and approval was not required for the study on human participants in accordance with the local legislation and institutional requirements. The patients/participants provided their written informed consent to participate in this study. Written informed consent was obtained from the individual(s) for the publication of any potentially identifiable images or data included in this article.

## Author Contributions

PH-B assessed, treated, and followed-up with the patient. AR interviewed the patient, conceptualized the case report, drafted the manuscript, and developed all figures. LKJ and SC contributed to the literature review and assisted with manuscript preparation. LG, QT, and MR provided substantial contributions to the interpretation of data as well as manuscript revisions. All authors have read and approved the final manuscript.

## Conflict of Interest

The authors declare that the research was conducted in the absence of any commercial or financial relationships that could be construed as a potential conflict of interest.

## Publisher's Note

All claims expressed in this article are solely those of the authors and do not necessarily represent those of their affiliated organizations, or those of the publisher, the editors and the reviewers. Any product that may be evaluated in this article, or claim that may be made by its manufacturer, is not guaranteed or endorsed by the publisher.

## Abbreviations

BMI: body mass index
BN: bulimia nervosa
DSM-5: diagnostics and statistical manual of mental disorders, 5th edition
EDE-Q: eating disorder examination questionnaire
KAP: ketamine assisted psychotherapy
NMDAr: N-methyl-D-aspartate receptor
PBS: pseudo-bartter's syndrome.
